
JOURNAL INFORMATION
==============================
NLM Title Abbreviation: Eur Psychiatry
Journal ID: Eur Psychiatry
NLM Journal ID: 9111820
Journal ID: EPA

European Psychiatry

ISSN: 0924-9338
EISSN: 1778-3585
Publisher: Cambridge University Press

ARTICLE INFORMATION
==============================
PMCID: PMC9412716
DOI: 10.1192/j.eurpsy.2020.9
Article ID: S0924933820000097
Article version: 1
Subjects: Abstracts

Plenary

Publication date: 2020 Jul
Electronic publication date: 2020 Sep 4
Volume: 63
Issue: Suppl 1
Issue title: Abstracts of the 28th European Congress of Psychiatry
First page: S596
Last page: S596
Copyright: © The Author(s) 2020
Copyright year: 2020
Copyright holder: The Author(s)
License: This is an Open Access article, distributed under the terms of the Creative Commons Attribution licence (http://creativecommons.org/licenses/by/4.0/), which permits unrestricted re-use, distribution, and reproduction in any medium, provided the original work is properly cited.
License URL: https://creativecommons.org/licenses/by/4.0/

Page count: 1

==============================

Article ID: PL002
Subjects: The Stepchild of Medicine: Psychiatry's Notorious Past and Bright Future

The notorious past and bright future of psychiatry

Lieberman J.
Columbia University , Psychiatry, New York , United States of America

Conflict of interest:

No

Article ID: PL003
Subjects: Psychopathology 2020: The Heritage of Karl Jaspers

Psychopathology 2020: the heritage of karl jaspers

Maj M.
University of Campania L. Vanvitelli , Department of Psychiatry, Naples , Italy

Conflict of interest:

No

